# The Seeds of Doubt: Finding Seeds in Intriguing Places

**DOI:** 10.3389/fmed.2021.655113

**Published:** 2021-04-14

**Authors:** Federica Grillo, Michela Campora, Laura Cornara, Alberta Cascini, Simona Pigozzi, Paola Migliora, Francesca Sarocchi, Luca Mastracci

**Affiliations:** ^1^Pathology Unit, Department of Surgical and Diagnostic Sciences (DISC), University of Genoa, Genoa, Italy; ^2^Ospedale Policlinico San Martino, Genoa, Italy; ^3^Department for the Earth, Environment and Life Sciences (DiSTAV), University of Genoa, Genoa, Italy; ^4^Clinical Cytopathology Service and Pathology Institute of Locarno, Locarno, Switzerland; ^5^Institute of Pathology, Medical University of Graz, Graz, Austria

**Keywords:** seeds, gastrointestinal pathology, appendicitis, diverticular disease, endoscopic biopsy, polipectomy

## Abstract

**Introduction:** Seeds may be found in gastrointestinal tissue samples, and their multifaceted appearance may be challenging. The aim is to report a rough incidence of pathology samples which show seeds, specify the most frequent sample types and show an iconography of the most commonly identified seeds.

**Materials and Methods:** Between 2017 and 2020, all gastrointestinal pathology cases in which seeds/seed parts were found, were collected and seed type described by referencing a seed image library.

**Results:** Fifty cases with complete seeds/seed parts were collected: 16 colonic resections for colorectal cancer and diverticulosis, 13 appendiceal resections for appendicitis, 1 gastric resection. Fifteen cases were found in polypectomy specimens and 5 cases in colorectal endoscopic biopsies. Most frequent seed types were tomato, kiwi, blueberry, and blackberry seeds.

**Conclusion:** Seeds may be found in up to 4% of specimens; their recognition may be useful to exclude parasitic infections as well as in forensic sciences.

## Introduction

Gastrointestinal pathology specimens make up a large part of the day to day practice of any busy pathology practice with specimens ranging from small endoscopic biopsy samples to major surgical resections. In most cases, alimentary matter is not clearly visible as bowel preparation effectively cleans the gut before endoscopy or surgical specimens are washed of feces after opening. Rarely, at microscopy, the pathologist encounters “alien” material in the bowel lumen which may cause some difficulties in recognition. In particular, plant matter, such as seeds, may prove to be resistant to digestion and found in the bowel lumen where their multifaceted appearance may be challenging for histopathologists, with regards to recognition and differential diagnosis.

In our daily practice we have noticed an increase in the incidental finding of undigested seeds and vegetable residues in gastrointestinal specimens. Recent promotion of a more vegetarian diet, including varied fruit and seed consumption, for their known fiber content and anti-oxidant properties, may partly explains this observation. The whole of the gastrointestinal tract is a possible site for sampling and for seed/plant recognition and pathologists may often encounter such matter, while rarely being able to precisely identify the seed/plant matter type. This specific seed identification may however prove important in some instances, such as in forensic pathology, where evidence of a victim's last meal may prove useful.

In 2016 we observed some seed samples in appendixes, resected for appendicitis, and tried to identify seed type by contacting our academic forensic botanist (1). This led to a fruitful collaboration which turned out to be rather fun. With this in mind we have been collecting, from our regular gastrointestinal pathology sign out cases, all samples which present with incidental seeds, identifying pathology sample type, disease and, where possible, seed type. The aim is to report a rough incidence of cases which show seeds in pathology samples, specify the most frequent sample types and show a clear iconography of the most commonly identified seeds.

## Materials and Methods

Starting from June 2017 to June 2020, all gastrointestinal pathology cases in which seeds or seed parts were found, were prospectively collected. The following data were registered: (1) patient related details such as gender and age, (2) sample site, (3) sample type (e.g., endoscopic biopsy, endoscopic polypectomy, surgical resection etc), (4) underlying disease, (5) seed type.

Seed type was recognized by comparison with a reference image library of seeds and plant matter which have been formalin fixed, paraffin embedded, microtome cut (4-micron thick sections) and stained with Haematoxylin and Eosin (1, 2). Up to now almost 70 different alimentary seeds have been categorized and photographed.

## Results

A total of 50 cases from 50 patients with complete seeds or seed parts were collected.

Twenty-nine patients were female and 21 were male; mean age was 51 years (range from 15 to 92 years). With regards to site, most samples came from the large intestine (32 cases, 64%), followed by the caecal appendix (13 cases, 26%), small intestine (4 cases, 8%) and stomach (1 case, 2%).

**Figure 1:**
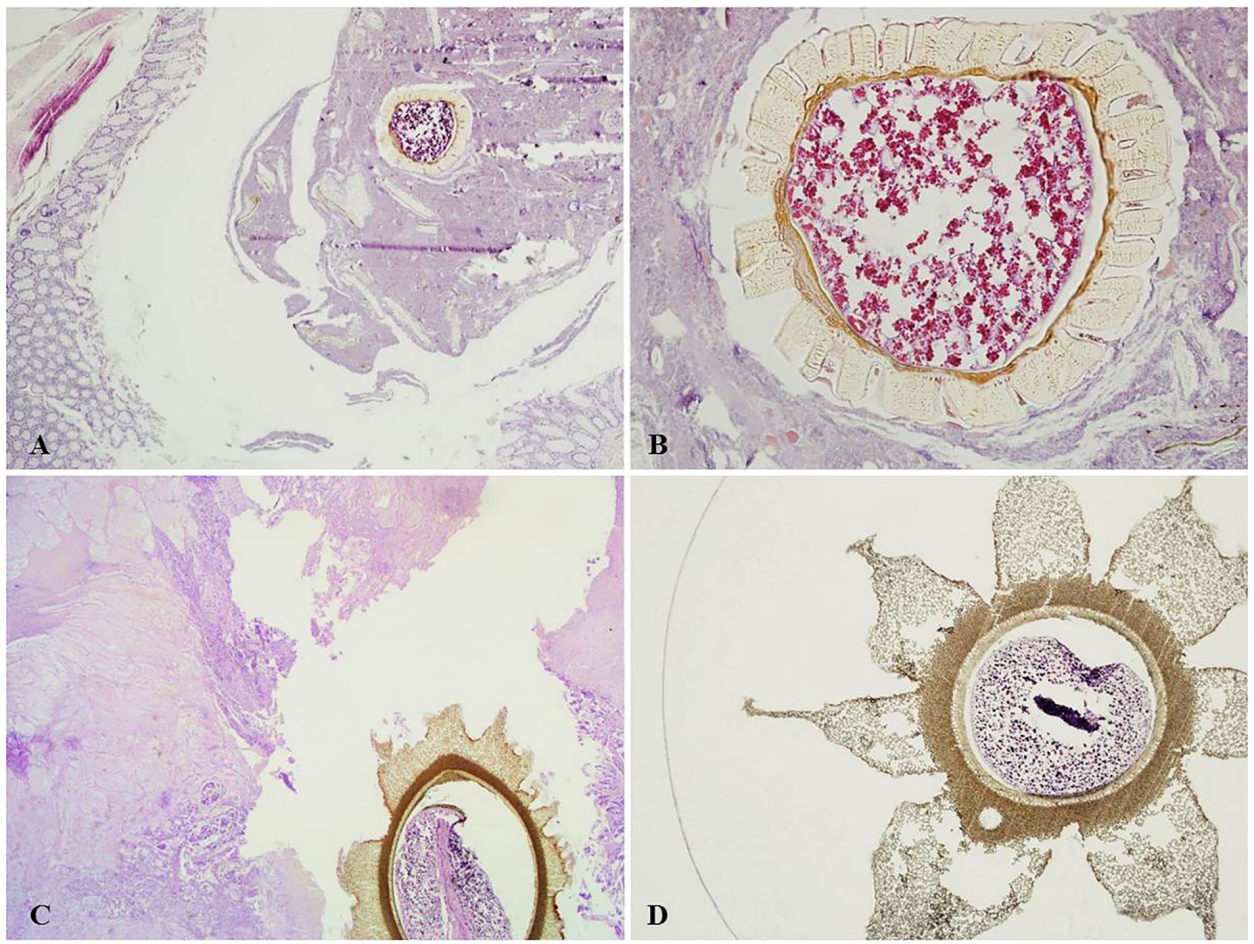
**(A)** Blueberry seed within colic diverticulum with no inflammation (H&E, magnification 4x). **(B)** Blueberry seed in **(A)**, showing partly preserved starch granules in the endosperm; the seed coat shows evenly thickened inner and radial U-shaped walls, with many pores (H&E, magnification 20x). **(C)** Papaya seed adherent to the ulcer base of colorectal adenocarcinoma (H&E, magnification 2x). **(D)** Magnification of a papaya seed showing the characteristic star-shaped outer mesotesta (H&E magnification, 4x).

**Figure 2:**
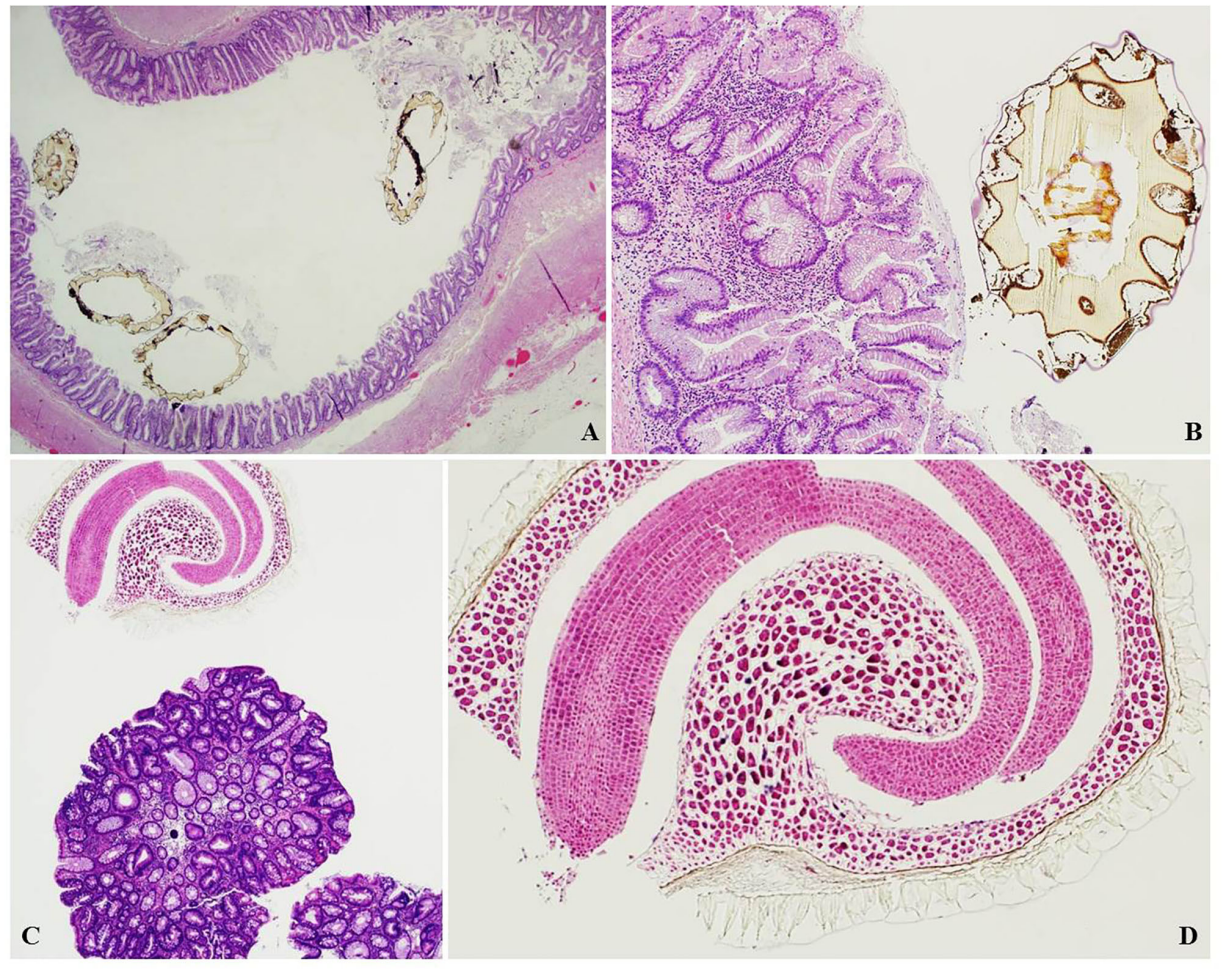
**(A)** Kiwi seeds within a distended appendix with serrated lesion (H&E, magnification 2x). **(B)** Magnification of the kiwi seed in **(A)**, showing the thick, scalloped seed coat (H&E, magnification 10x). **(C)** Goji seed found in an endoscopic polypectomy specimen for colorectal tubular adenoma (H&E, magnification 4x). **(D)** Magnification of a goji seed showing characteristic curved embryo and endosperm (H&E magnification, 10x).

Most seeds were found in surgical specimens (total of 30 cases -60%) of which 16 cases were found in colonic resections (32%), 13 cases in appendiceal resections (26%) and 1 in a gastric resection (2%) (Figures 1, 2). Considering the 16 colic resections, 7 were due to colorectal cancer, 7 in diverticular disease, 1 due to intestinal infarction and 1 due to ileo-colic Crohn's disease. Of the 13 appendicectomies, 11 were for obstructive appendicopathy, 1 due to acute appendicitis, and 1 due to low grade appendiceal mucinous neoplasm. The single gastrectomy case was performed for invasive gastric cancer.

**Figure 3:**
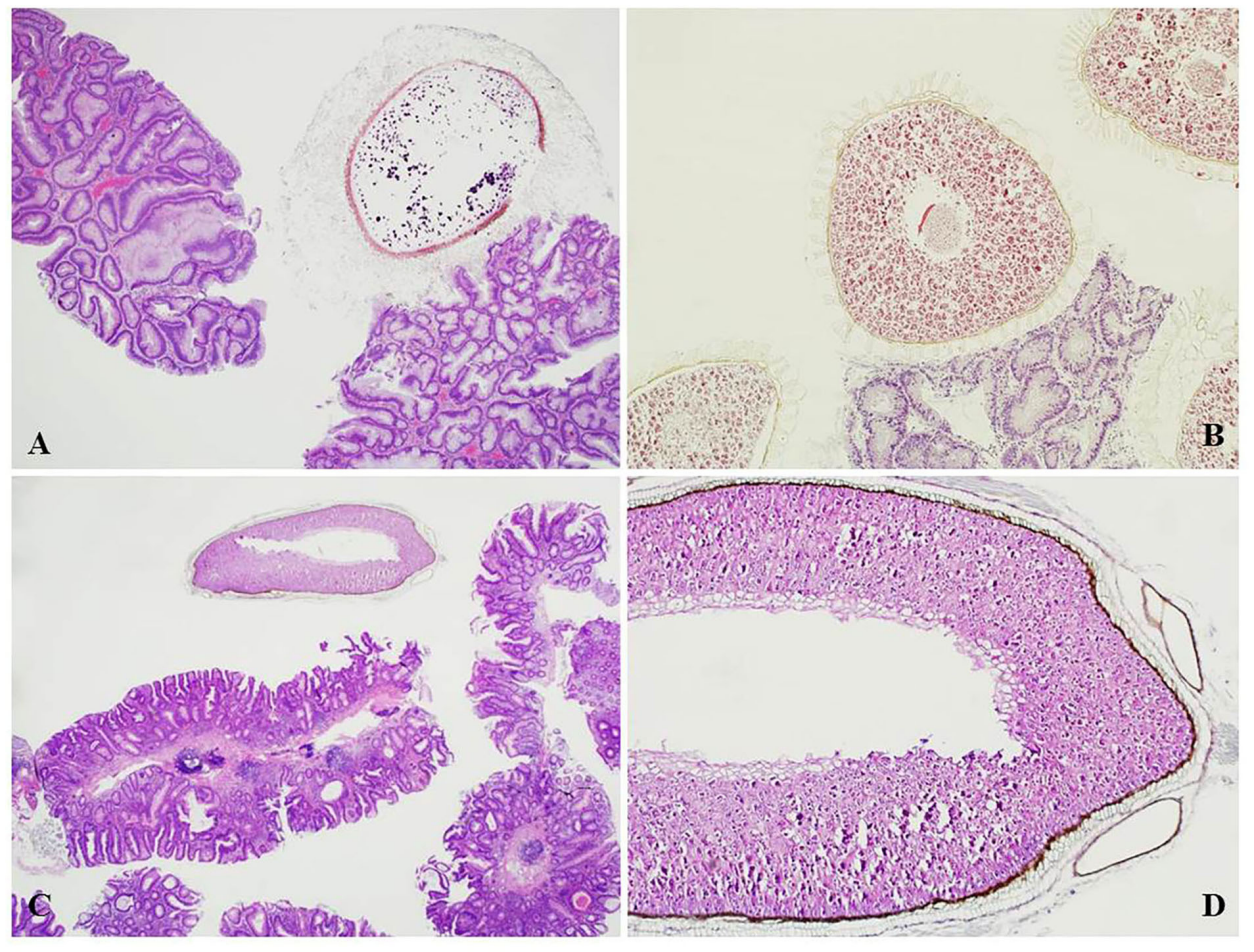
**(A)** Tomato seed found adherent to a colorectal tubulo-villous adenoma with seed coat hairs attached to the irregular surface of the polyp (H&E, magnification 4x). **(B)** Blueberry seeds in an endoscopic polypectomy specimen showing similar attachment of the seed coat to the surface area of a tubular adenoma (H&E, magnification 10x). **(C)** Seed of uncertain type in an endoscopic polypectomy specimen for colorectal tubular adenoma (H&E, magnification 4x). **(D)** Magnification of the seed in **(C)** showing starch granules (H&E magnification, 10x).

A further 30% (15 cases) were found in endoscopic polypectomy specimens for colorectal adenoma and 10% (5 cases) in colorectal endoscopic biopsies for altered bowel habits (Figures 2, 3).

With regards to rough incidence of seed retrieval in the most frequent samples, seeds were found in 2.2% of appendicectomy specimens (13/588 cases), in 4.1% of colic resections for diverticular disease (7/169 cases), 0.9% of colorectal carcinoma resections (7/757 cases), 0.7% of endoscopic polypectomies (15/2,076 cases), and 0.3% of endoscopic biopsy specimens (5/1,642 cases) over the same period of time.

Most frequent seed types were tomato seeds or parts in 12 cases (24%), followed by kiwi seeds in 11 cases (22%), blueberry seeds in 6 cases (12%), and blackberry seeds in 4 cases (8%). Rare seed types were seen in 8 cases such as millet, chia, papaya, sesame, and goji. In 10 cases seeds were identified, but it was not possible to classify them with certainty as they were fragmented or not part of our seed library. Seed types did not show a predilection for either sample site, type nor underlying disease.

## Discussion and Conclusions

Our diet is rich in vegetables and fruits, many of which have small seeds with hard and impermeable seed coats. In nature, endozoochorous seed dispersal, based on a mutualistic interaction between plants and animals, is a very common mechanism of seed dispersion, frequently causing seed coat abrasion which ensures a better chance of seed germination. However, the integrity of the seed coat must be at least partly preserved to protect the delicate embryo during its passage through the animal's digestive system. Seeds present within edible fruits and vegetable are generally ingested without mastication, so that they pass almost intact through the intestine. Previous studies (1) have shown that no significant difference in morphology is detectable between undigested and pseudo-digested seeds, e.g., seeds pretreated to simulate the action of gastric acids and bile, so that many seeds can reach the large intestine almost intact. It is not surprising, therefore, that in human pathology samples, undigested seeds can be sometimes found adhering to the gastrointestinal tract. In addition, peel, leaf fragments, or other indigestible parts of plant foods can resist digestion and may therefore be found in histology sections, for example, leaf and fruit cuticles rich in waxes and cutin (2).

Reasons which may explain why most seeds were found in only a few types of samples include: (1) embedding of seeds in anatomic crevices such as the appendix or in diverticulae (3); (2) seeds may become entangled on the villous surface of adenomas or on the necrotic bed of ulcerated cancers; (3) seeds may cling to the bowel wall, even after bowel preparation, due to an irregular or gelatinous outer layer which increases adherence. Furthermore, the relatively high incidence of seed identification in resections for diverticular disease (4% of cases) or in appendicectomy specimens (2.2% of cases) is probably, in part, related to the acute onset of symptoms leading to surgery, for which seeds may be directly responsible, and in part to the fact that seeds become wedged in places which are difficult to emerge from.

While some seed types are readily recognized as such, others may show a perplexing conformation which may resemble helminthes or parasitic infections. Indeed, 3 of the cases we collected were sent as referrals as the submitting pathologists were in doubt whether these could be some un-named/unrecognized worm. In most cases, the presence of starch granules and the thick cutinous surface coat leads to correct interpretation.

Identifying the *precise* seed type may become fundamental in forensic science where plant matter identification may greatly contribute to crime solving. Forensic botany is the application of plant sciences to criminal investigations (4) as well as in toxicology and other non-criminal settings (5). Plant parts may be found entrapped on the victim's clothes or as partially digested or undigested fragments in gastric/bowel contents, aiding in the identification of time of death, last meal or which path was taken by the victim before body discovery (6). While DNA analysis and barcoding are available, the forensic pathologist may also be required to identify and characterize plant fragments on histology slides (7). Unfortunately, there is a dearth of useful microscopic iconography available to help in the identification of different seeds and other plant remnants. Most botanical texts show drawings of seeds, mainly referring to external morphologic features, that are often difficult to access in the medical literature or understand by pathologists. Furthermore, it may prove difficult to obtain complete images of seeds as the thickened seed coat makes processing, paraffin embedding and microtome sectioning particularly difficult, thereby losing the innermost part of the seed.

In conclusion, seeds and plant fragments may be found in the gut lumen creating possible diagnostic conundrums, however, as the 19th century naturalist Richard Jefferies once wrote, “If every plant and flower were found in all places the charm of locality would not exist. Everything varies, and that gives the interest.”

## Data Availability Statement

The raw data supporting the conclusions of this article will be made available by the authors, without undue reservation.

## Author Contributions

FG, MC, LC, and LM designed the study, collected samples, and wrote the paper. SP and AC performed technical analysis and organized the seed library collection. PM and FS contributed in collecting and identifying seeds. All authors contributed to the article and approved the submitted version.

## Conflict of Interest

The authors declare that the research was conducted in the absence of any commercial or financial relationships that could be construed as a potential conflict of interest.
